# Knowledge‐distilled diffusion models for improving cone‐beam CT image quality with meta‐learning under imbalanced data

**DOI:** 10.1002/mp.70530

**Published:** 2026-07-31

**Authors:** Joonil Hwang, Sangjoon Park, Seungryong Cho, Jin Sung Kim

**Affiliations:** ^1^ Department of Radiation Oncology, Yonsei Cancer Center, Heavy Ion Therapy Research Institute Yonsei University College of Medicine Seoul South Korea; ^2^ Yonsei Institute for Digital Health Yonsei University Seoul South Korea; ^3^ Department of Nuclear and Quantum Engineering KAIST Daejeon South Korea; ^4^ Medical Image and Radiotherapy Lab (MIRLAB) KAIST Daejeon South Korea; ^5^ Oncosoft Seoul South Korea; ^6^ Medical Physics and Biomedical Engineering Lab (MPBEL) Yonsei University College of Medicine Seoul South Korea

**Keywords:** cone‐beam computed tomography, diffusion model, knowledge distillation, meta‐guidance

## Abstract

**Background:**

Adaptive radiation therapy (ART) relies on daily cone‐beam CT (CBCT), yet its limited image quality hinders accurate dose calculation, particularly under substantial anatomical changes.

**Purpose:**

To overcome the clinical challenge of scarce paired planning CT (pCT) data versus abundant unpaired CBCTs, we propose a framework driven by two core components: knowledge distillation and gradient‐based meta‐guidance.

**Methods:**

The knowledge distillation strategy enables the model to leverage the vast unpaired dataset. Complementing this, the meta‐guidance mechanism stabilizes training by dynamically updating the weight of each unpaired sample; it assigns higher importance to pseudo‐labels that align with trusted supervised gradients, effectively filtering out noise. Our method was evaluated on a cohort of 99 breast cancer patients (with 19 reserved for testing) and further evaluated on a public dataset to assess the generalization capability.

**Results:**

The proposed approach demonstrated superior performance, achieving the best quantitative metrics (MAE 13.22 HU, SSIM 0.9516, PSNR 30.35 dB). It significantly outperformed representative supervised, unsupervised, and standard distillation baselines (p<0.01). Ablation studies confirm that our method enhances image quality while preserving the patient's daily anatomy by minimizing feature hallucination.

**Conclusions:**

By uniquely combining knowledge distillation with meta‐guidance, our method advances the frontier of high‐quality synthetic CT, enabling more robust and adaptive ART workflows.

## 1 INTRODUCTION

Cone‐beam computed tomography (CBCT) is routinely employed in adaptive radiation therapy (ART) to account for daily anatomical variations. While ART enhances tumor dose conformity and spares organs at risk (OARs), the inherently inferior image quality of CBCT limits its direct utility for precise dose calculation. This limitation is particularly pronounced when anatomical deformations are significant, often due to tumor response or weight loss—since deformable image registration (DIR) between a planning CT (pCT) and a daily CBCT can introduce substantial inaccuracies. Such errors are frequently reported in low‐contrast regions like the pelvis and abdomen.^1^, ^2^ Similarly, in breast cancer radiotherapy, where targets and OARs span the complex thoracic anatomy (e.g., chest wall, lung interface, and heart) and surgical clips are prevalent, faithful Hounsfield Unit (HU) recovery and artifact suppression are especially critical for ensuring accurate dose computation and safe online adaptation.

**FIGURE 1:**
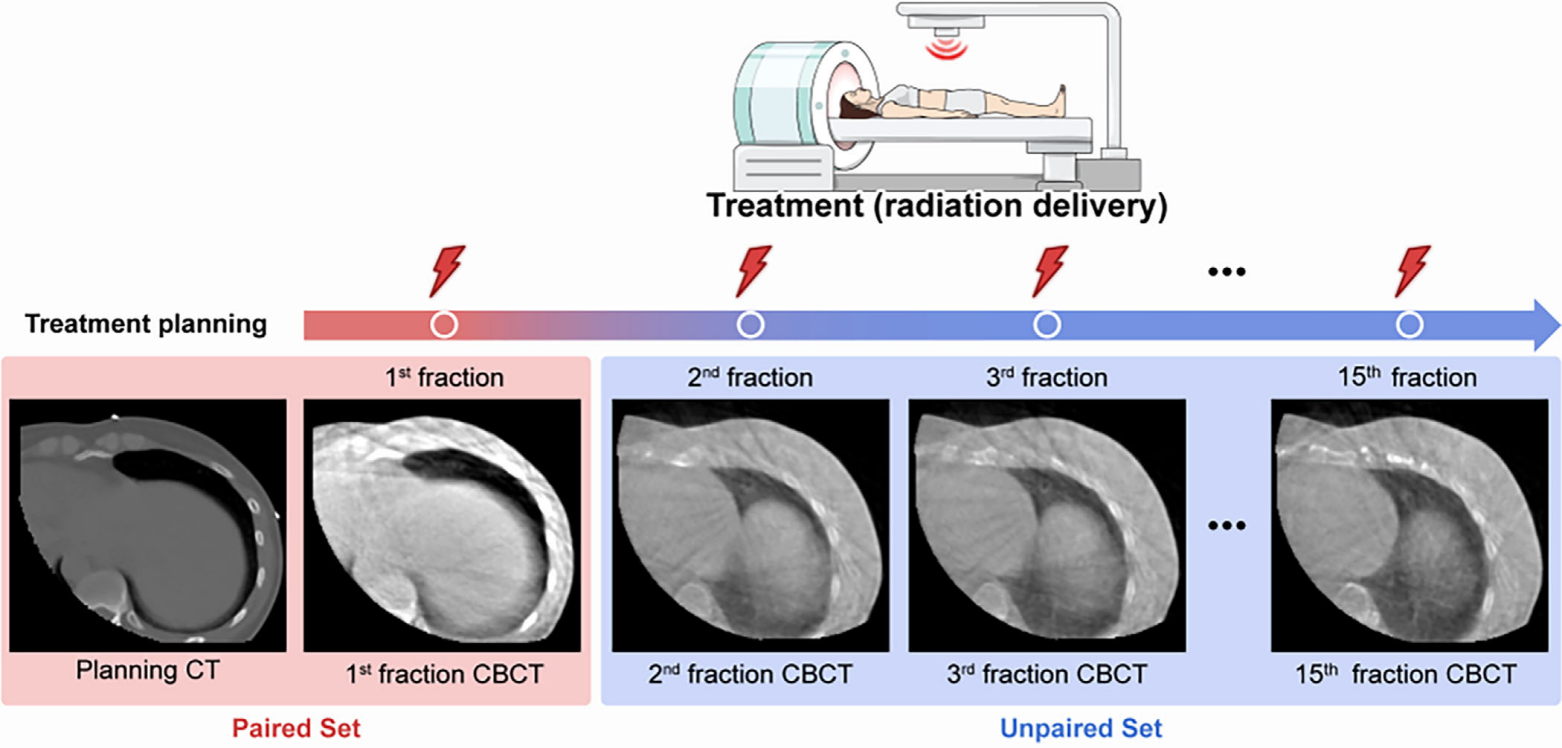
Schematic overview of the clinical data acquisition timeline in breast cancer ART. The **Paired Set** (red bracket) is formed by the pCT and the 1^st^ fraction CBCT due to their temporal proximity. In contrast, the **Unpaired Set** (blue bracket) consists of the abundant daily CBCTs acquired from subsequent fractions (e.g., 2^nd^–15^th^), for which no corresponding same‐day pCT exists. This distinct separation highlights the severe “few‐paired vs. many‐unpaired” data imbalance that motivates our proposed knowledge‐distilled and meta‐guided diffusion framework.

To mitigate these limitations, significant research has focused on generating synthetic CT (sCT) from CBCT.^3^, ^4^ However, applying standard deep learning methods faces a fundamental challenge in the clinical setting: severe data imbalance and imperfect pairing. In practice, perfectly paired pCT–CBCT data are rare because (i) several days or more typically elapse between pCT acquisition and treatment start, (ii) same‐day acquisition of pCT and CBCT is uncommon in routine workflows, and (iii) anatomy evolves over the treatment course (e.g., tumor shrinkage, edema resolution, and weight change), undermining exact spatial correspondence even when patient identity matches. Furthermore, while a pCT is typically acquired only 1–3 times during a treatment course, a CBCT is acquired daily for image‐guided radiation therapy (IGRT), resulting in 10–30 scans depending on the general clinical protocol. In our specific institutional breast cancer cohort, a 15‐fraction protocol was employed. As summarized in Figure 1, this clinical workflow creates a distinct separation between the sparse **Paired Set** (1^st^ fraction) and the abundant **Unpaired Set** (2^nd^–15^th^ fractions), highlighting the severe “few‐paired vs. many‐unpaired” imbalance. Consequently, purely supervised approaches are constrained by the scarcity of high‐quality paired data and fail to leverage the vast majority of available unpaired CBCTs.

To overcome the lack of paired data, several studies have explored unsupervised style transfer methods like CycleGAN^5^, ^6^ and MUNIT.^7^ While capable of learning from unpaired sets, these indirect approaches often struggle with clinical requirements, introducing structural inaccuracies or distorting CT HU values, which is a critical limitation for dose calculation.^8^

In contrast, diffusion‐based models have recently demonstrated remarkable potential in medical image synthesis tasks, offering high‐fidelity reconstruction and stable training behavior.^9^, ^10^ Nevertheless, their adoption in radiotherapy applications remains limited, primarily because these models are inherently designed under a supervised learning paradigm that relies on paired training data. This dependence poses a substantial challenge in radiotherapy settings, where severe data imbalance exists.

In this work, we propose a novel framework to address these challenges. Our strategy is twofold: first, to leverage the vast amount of unpaired CBCT data by generating pseudo‐labels through knowledge distillation; and second, to employ a gradient‐based meta‐guidance mechanism that selectively utilizes valid learning signals from these pseudo‐labels to stabilize model updates. Through this approach, we aim to answer a critical question: *“How can a model effectively perform knowledge distillation in a label‐scarce environment while preventing performance degradation caused by the inherent noise in pseudo‐labels?”*

A preliminary version of related work was presented at MICCAI 2024.^11^ While the conference version introduced the fundamental concept of using knowledge distillation with diffusion models for imbalanced data, this extended manuscript significantly expands the methodology by introducing a novel “Gradient‐Based Meta‐Guidance” mechanism. This mechanism stabilizes the self‐training process by filtering out noisy pseudo‐labels via gradient alignment, which was not present in the preliminary version. Furthermore, we have added a rigorous data efficiency analysis and validated the model's generalization capability on a large external public dataset (SynthRad 2025).

Inspired by recent advances in knowledge distillation and self‐training,^12^, ^13^ our framework enforces consistency between supervised gradient signals and the pseudo‐supervised learning pathway, ensuring more robust and reliable optimization.

We implement our framework using a diffusion model backbone and demonstrate through extensive experiments that our approach surpasses traditional supervised and unsupervised baselines in structural similarity and tissue density accuracy. By uniquely combining knowledge distillation with meta‐guidance, our method advances the frontier of high‐quality pCT synthesis, enabling more robust and adaptive ART workflows.

## 2 METHODOLOGY

### 2.1 Dataset and Study Design

We analyzed a cohort of 99 breast cancer patients who underwent adjuvant radiotherapy at a single institution. Each patient had a pCT and daily CBCT acquired during treatment. To reflect real‐world acquisition scenarios and enable robust model deployment, the cohort was structured into two distinct groups to mimic a realistic clinical deployment scenario: a **legacy cohort** (n=89, strictly split into 70 for training and 19 for testing) and a **new cohort** (n=10 with a complete course, fractions 1^st^–15^th^).

**FIGURE 2:**
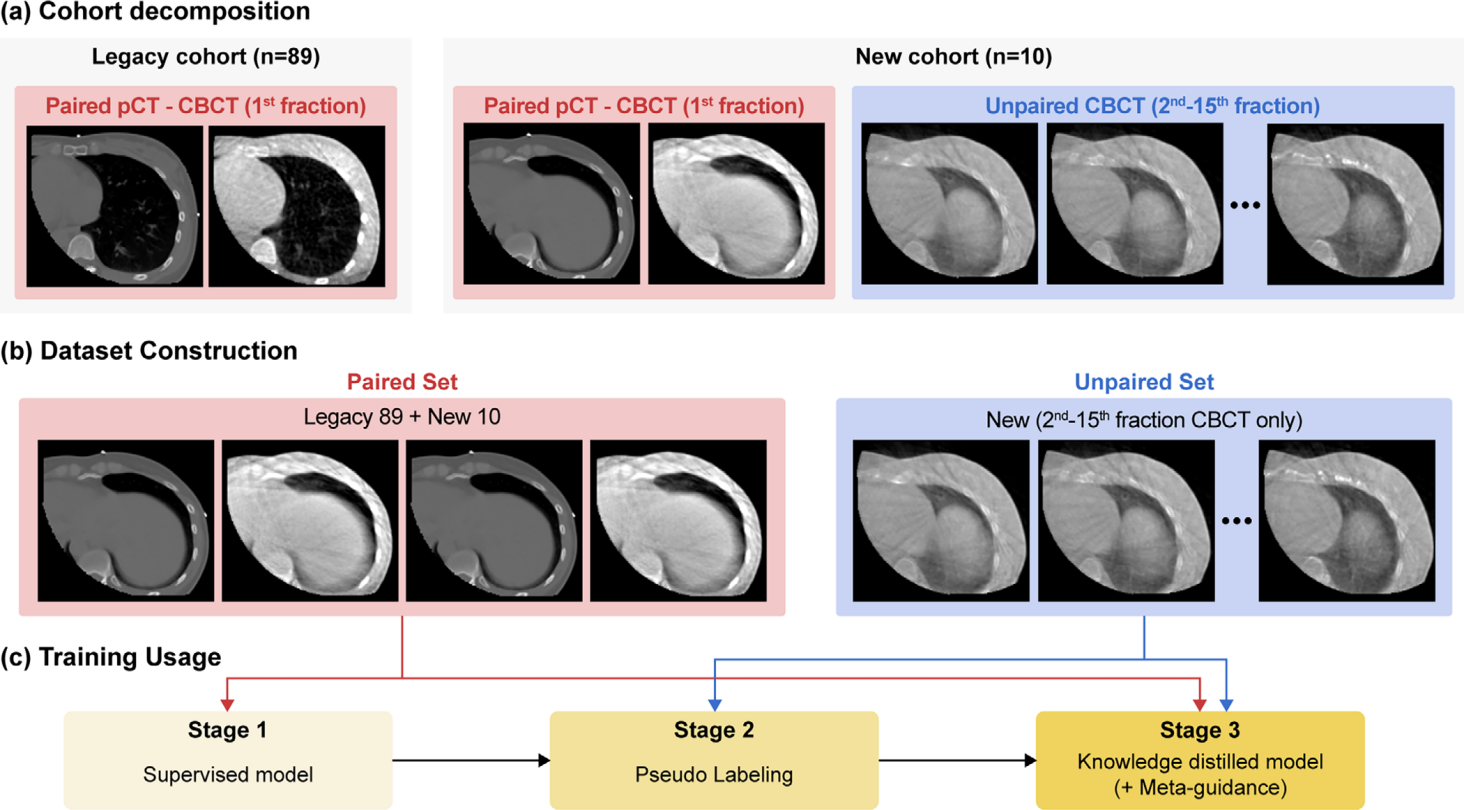
Overview of the cohort decomposition and training framework. (a) **Cohort decomposition**: The study utilizes a legacy cohort (n=89) with paired data and a new cohort (n=10) containing both paired (1^st^ fraction) and unpaired (2^nd^–15^th^ fractions) data. (b) **Dataset Construction**: The *Paired Set* (red outlines) serves as the ground truth source, while the *Unpaired Set* (blue outlines) simulates daily clinical data. (c) **Training Usage**: The pipeline progresses from a supervised model (Stage 1) to pseudo‐labeling (Stage 2), culminating in the proposed knowledge‐distilled model trained with meta‐guidance (Stage 3).

Designed to address the “few‐paired vs. many‐unpaired” imbalance described in the Introduction, our study utilizes a three‐stage pipeline instantiated in Figure 2. This schema visually details how we leverage the **legacy Cohort** for supervised training and the **new Cohort** to simulate the real‐world scenario of unpaired daily acquisitions. We constructed two distinct datasets based on the acquisition timeline:

- **Paired Set** (indicated in red): This set comprises the entire legacy cohort (n=89) plus the 1^st^ fraction pCT–CBCT pairs from the 10 patients in the new cohort. The 1^st^ fraction is selected as the ground truth because it is acquired at the start of treatment, exhibiting minimal anatomical deviation from the pCT. Crucially, for the 10 patients in the new cohort, this 1^st^ fraction paired data serves as the indispensable patient‐specific reference (anchor) required to compute the gradient‐based meta‐guidance weights when learning from their subsequent unpaired fractions in Stage 3.
- **Unpaired Set** (indicated in blue): This set consists of CBCT scans from the 2^nd^–15^th^ fractions of the 10 patients in the new cohort. The corresponding pCTs were intentionally withheld to simulate the abundant stream of unpaired daily imaging encountered in clinical practice.

The overall training workflow (Figure 2c) proceeds as follows: *Stage 1* trains a supervised model exclusively on the Paired Set; *Stage 2* utilizes this supervised model to generate pseudo‐label sCTs for the Unpaired CBCTs; and *Stage 3* trains the final knowledge‐distilled model on the combined (Paired + Pseudo‐paired) data, stabilized by our weight update meta‐guidance mechanism.

To eliminate any ambiguity regarding the cohort decomposition and to explicitly confirm strict patient‐wise separation, we summarize the exact data splits and their utilization across the training and evaluation stages in Table 1. Notably, the 80‐patient paired dataset used for baseline comparisons consists of the 70 patients in the legacy training cohort and the 1^st^ fractions of the 10 patients in the new cohort. The 19 test patients are strictly isolated from all training procedures.

**TABLE 1:** Summary of cohort decomposition and data utilization across training stages and evaluation.

Cohort split	Data description	Usage in framework
**Legacy train** (*n* = 70)	Paired pCT and CBCT	**Stage 1**: Supervised teacher training **Stage 3**: Distillation and meta‐guidance **Baselines**: Supervised baseline training
**New cohort** (*n* = 10)	**1** ^**st**^ **fraction**: Paired pCT/CBCT **2** ^**nd**^ **–15** ^**th**^ **fractions**: Unpaired CBCTs	**Stage 2**: Pseudo‐label generation (using unpaired) **Stage 3**: Meta‐guidance anchor (using 1^st^ fraction) and distillation (using unpaired) **Baselines**: 1^st^ fraction added to 70 legacy cohort (forming the 80‐ patient paired set)
**Legacy test** (*n* = 19)	Paired pCT and CBCT	**Evaluation only**: Strictly held‐out test set for final performance metrics.

*Note*: Strict patient‐wise separation is maintained for the held‐out test set.

### 2.2 Image Acquisition

Planning CTs were acquired using a TOSHIBA scanner (120 kVp, in‐plane matrix 512×512, voxel size 1.367×1.367×3 ${\mathrm{mm}}^{3}$), while daily CBCTs were obtained using an ELEKTA XVI system (100 kVp) with an identical reconstruction matrix and voxel spacing. Given the context of breast and chest irradiation, where targets and OARs span complex thoracic anatomy (e.g., chest wall, lung interface, and heart) and metallic clips are frequently present, ensuring faithful HU recovery and artifact suppression is critical.

### 2.3 Preprocessing and Registration

To establish accurate spatial correspondence for paired training samples, we corrected residual misalignments using a rigorous two‐stage registration pipeline implemented via the MATLAB Image Processing Toolbox (MathWorks, Natick, Massachusetts, USA). Initially, a global 3D translation was applied to correct bulk anatomical misalignments between the pCT and the corresponding 1^st^ fraction CBCT. This rigid step was driven by the Mattes Mutual Information metric and optimized using a One‐Plus‐One Evolutionary algorithm (initial radius = 0.004) to robustly handle multimodal intensity differences. Following this alignment, the volumes were cropped longitudinally to match the valid field of view (FOV) of the CBCT to a 256×256 axial region to exclude extraneous structures and focus on the patient's core anatomy. Subsequently, a non‐rigid registration was performed using a displacement‐field‐based deformable image registration algorithm (imregdeform). This approach computes a dense displacement vector field driven by intensity‐based **Sum of squared differences (SSD)** forces. To capture anatomical variations across multiple scales, we utilized a 3‐level multi‐resolution image pyramid (coarse‐to‐fine strategy). For regularization, a Gaussian smoothing kernel was applied to the accumulated displacement field at each iteration to maintain topological continuity and penalize unphysical deformations. For failure handling, all registered pairs were subjected to strict visual inspection for gross anatomical mismatches. To rigorously validate the topological sanity of these deformations and ensure they do not confound model training, detailed quality assurance (QA) including example overlays and Jacobian sanity statistics is provided in Supplementary Section S1. Before training, HU values were explicitly clipped to a clinically relevant global range of [−1000,3000] HU. These clipped intensities were then linearly normalized to a range of approximately [−1.0,1.5] to match the input distribution requirements of the diffusion model.

### 2.4 Ethics and IRB Approval

This retrospective study was approved by the Institutional Review Board at Yongin Severance Hospital (approval no. 4–2024–1174). The requirement for informed consent was waived due to the retrospective nature of the study and the use of anonymized data. All procedures adhered to relevant guidelines and regulations.

### 2.5 Overview of the Proposed Framework

**FIGURE 3:**
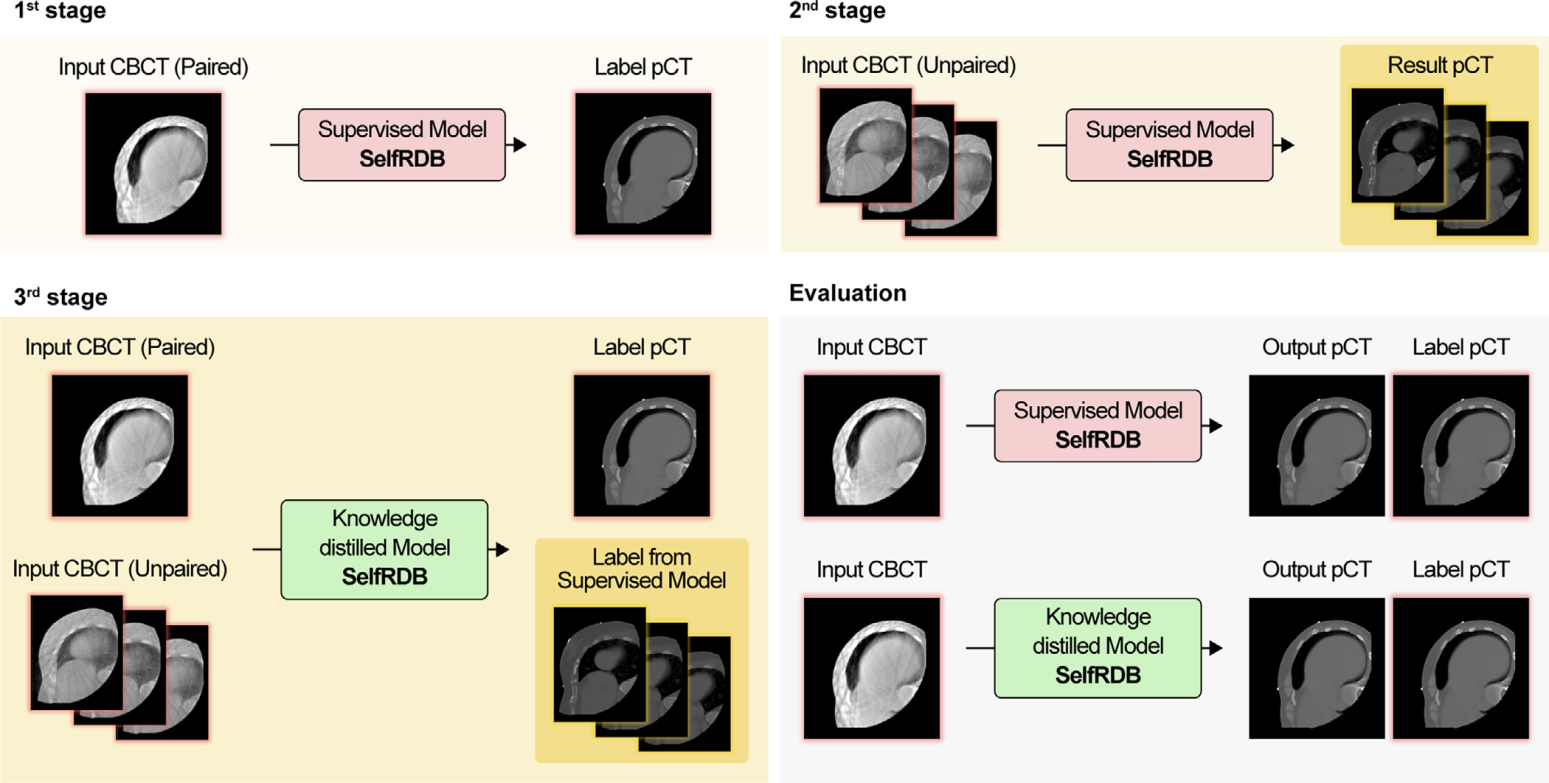
Schematic overview of the proposed training framework. The workflow proceeds as follows: **Supervised Training** establishes a baseline using paired data; **Pseudo‐Label Generation** creates synthetic targets for the abundant unpaired CBCTs using the trained supervised model; and **Knowledge Distillation** trains the final model on the combined dataset comprising both original paired data and newly generated pseudo‐paired data. The evaluation phase compares predictions from both the supervised and distilled models against ground truth pCTs on a held‐out test set.

Our framework proceeds in three stages, as schematically illustrated in Figure 3. This workflow is structured to progressively expand the training distribution from limited paired data to abundant unpaired data. We partitioned the 89 patients in the legacy cohort into a **training set** (n=70) and a **held‐out test set** (n=19). The 10 patients in the new cohort provide the longitudinal unpaired daily CBCTs (2^nd^–15^th^ fractions), while their 1^st^ fraction paired data are utilized exclusively to anchor the gradient‐based meta‐guidance mechanism.

- **Stage 1: Supervised Model Training With Self‐RDB**
  We train a supervised model, $\theta_{\text{sup}}^{*}$, *exclusively* on the **70 patients in the legacy training cohort** using their paired CBCT–pCT data. This step establishes a baseline mapping (CBCT→pCT) under full supervision.
- **Stage 2: Knowledge Distilled Model Training**
  The trained supervised model is applied to the **10 patients in the new cohort** unpaired daily CBCTs (2^nd^–15^th^ fractions) to synthesize corresponding sCTs. This process yields a **pseudo‐paired set** $\left\{x,\tilde{y}\right\}$ for these previously unusable fractions. Subsequently, we train the final knowledge‐distilled model, $\theta_{\text{distill}}$, on the union of three data sources: (i) the **70 legacy** paired samples, (ii) the **paired (**1^st^ **fraction)** data from the 10 patients in the new cohort, and (iii) the **pseudo‐paired** data $\left\{x,\tilde{y}\right\}$ derived from the 10 patients in the new cohort (2^nd^–15^th^ fractions).
- **Stage 3: Gradient‐Based Meta‐Guidance Post‐Training**
  This stage is stabilized by our gradient‐based meta‐guidance mechanism. Final evaluation is performed on the **19 held‐out legacy cohort**.

### 2.6 Stage 1: Supervised Model Training With Self‐RDB

In the first stage, we establish a supervised baseline using the paired dataset $D_{L}$ from the **70 legacy** patients (Figure 3, top‐left).

#### 2.6.1 Supervised Baseline Objective

The objective is to find the optimal parameters, $\theta_{\text{sup}}^{*}$, that minimize a standard supervised loss, $L_{\text{sup}}$ (e.g., $\ell_{1}$ distance):

(1) $$\theta_{\text{sup}}^{*}=\arg\min_{\theta_{\text{sup}}}E_{\left(x,y\right)\in D_{L}}\;\left[\;L_{\text{sup}}\left(f(x;\theta_{\text{sup}}),\;y\right)\right].$$

where $f(x;\theta_{\text{sup}})$ denotes the synthesized target image generated by the neural network parameterized by $\theta_{\text{sup}}$ given the input source image x.

#### 2.6.2 Network Architecture: Self‐Consistent Recursive Diffusion Bridge

**FIGURE 4:**
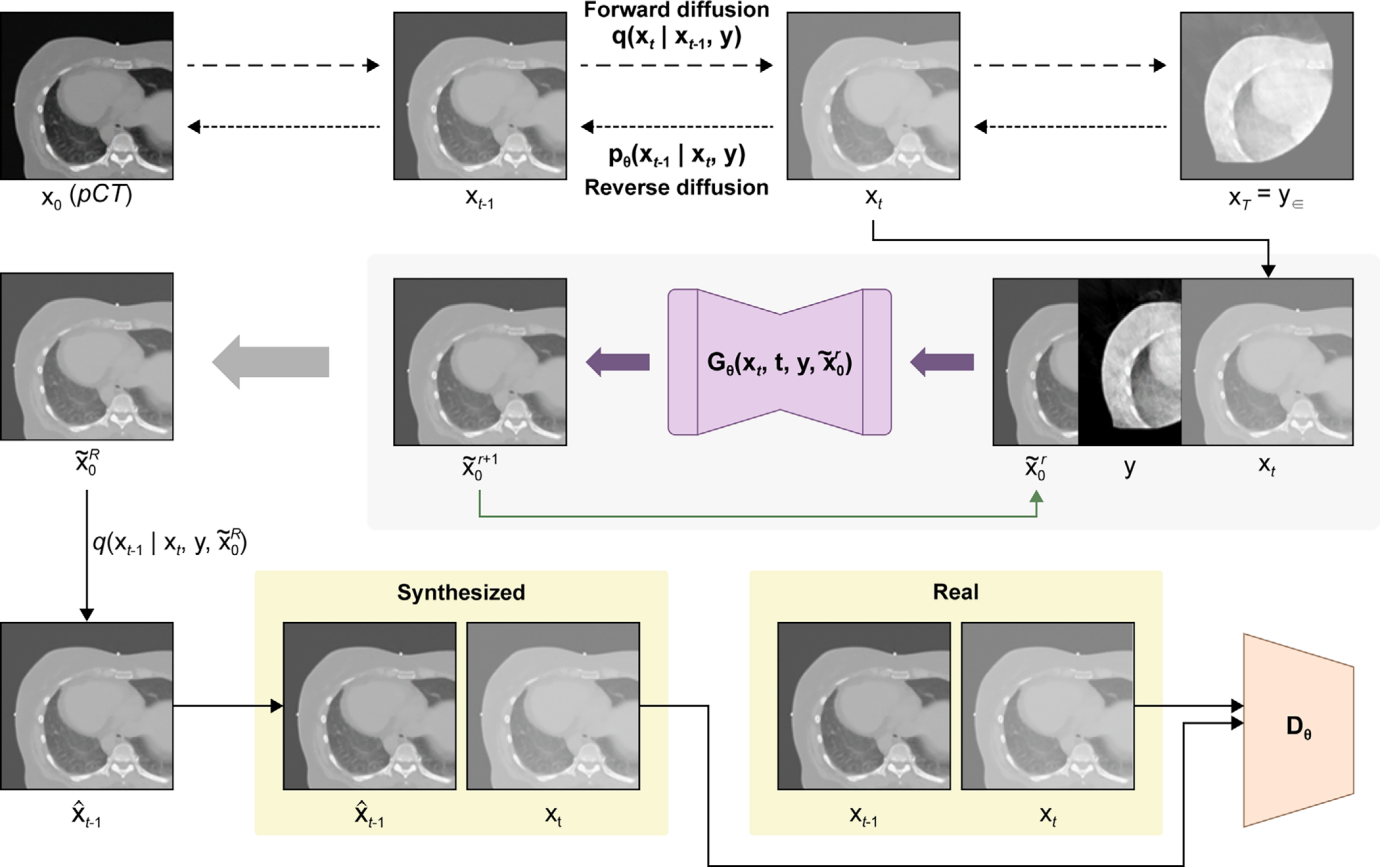
Overview of the Self‐RDB framework for CBCT→pCT translation.^14^ **Top**: The forward (bridge) process constructs a noisy state $x_{t}$ by mixing the target $x_{0}$ (pCT) and source y (CBCT) with scheduled noise, converging to $x_{T}\equiv y_{\varepsilon }$. **Center (gray panel)**: At each reverse step t, the generator $G_{\theta }$ recursively refines the clean estimate ${\tilde{x}}_{0}$ for R iterations (i.e., r=0⋯R−1) to yield a self‐consistent estimate ${\tilde{x}}_{0}^{*}$, which then guides the sampling process. **Bottom**: Posterior sampling produces the denoised state ${\hat{x}}_{t-1}$, while a discriminator $D_{\theta }$ provides adversarial feedback by distinguishing between real and synthesized transition pairs.

Our image translation backbone for this stage is the **Self‐Consistent Recursive Diffusion Bridge (Self‐RDB)**.^14^ As illustrated in Figure 4, this framework learns a mapping from a source image y∈Y (CBCT) to a target image $x_{0}\in X$ (pCT).

##### Forward (Bridge) Process

Unlike standard diffusion models, Self‐RDB constructs a stochastic bridge connecting $x_{0}$ and y. At timestep t, the state $x_{t}$ is defined as:

(2) $$x_{t}=\mu_{x_{0},t}\;x_{0}+\mu_{y,t}\;y+\sigma_{t}\varepsilon ,\;\varepsilon ∼N\left(0,I\right),$$

where T represents the final timestep of the forward diffusion process, such that the terminal state $x_{T}$ approximates a noisy version of the source $y_{\varepsilon }$.

##### Reverse Process with Self‐Consistency

Starting from $x_{T}\equiv y_{\varepsilon }$, the generator $G_{\theta }$ iteratively refines the estimated clean image ${\tilde{x}}_{0}$:

(3) $${\tilde{x}}_{0}^{\;r+1}=G_{\theta }\left(x_{t},t,y,{\tilde{x}}_{0}^{\;r}\right),\;r=0,\text{…},R-1,$$

until a self‐consistent estimate ${\tilde{x}}_{0}^{*}$ is achieved. The generator is optimized using a composite loss comprising an $\ell_{1}$ distance term and an adversarial term ($L_{G_{\theta }}$), while a discriminator $D_{\theta }$ is trained to distinguish real transitions from synthesized ones.

##### Adversarial Training Objective

To ensure both perceptual realism and pixel‐wise accuracy, the generator is optimized using a composite loss function comprising an $\ell_{1}$ distance term and an adversarial term:

(4) $$L_{G_{\theta }}=E\left[\lambda_{1}\;{∥x_{0}-{\tilde{x}}_{0}^{*}∥}_{1}-\log D_{\theta }\left({\hat{x}}_{t-1}\right)\right].$$

Simultaneously, a discriminator $D_{\theta }$ is trained to distinguish real transitions from synthesized ones, stabilized by a gradient penalty:

(5) $$\begin{array}{cc} L_{D_{\theta }} & =E\left[-\log D_{\theta }\left(x_{t-1}\right)-\log\left(1-D_{\theta }\left({\hat{x}}_{t-1}\right)\right)\right.\\ & \left.\;+\lambda_{2}\;{∥\nabla_{x_{t-1}}D_{\theta }\left(x_{t-1}\right)∥}_{2}^{2}\right]. \end{array}$$

In our experiments, we set the number of reverse steps T=10 and recursive refinements R=2 to balance reconstruction fidelity with computational efficiency.

### 2.7 Stage 2: Knowledge Distilled Model Training

Following the supervised training, we leverage unpaired data through a knowledge distillation process.

#### 2.7.1 Pseudo‐Label Generation

The supervised model $\theta_{\text{sup}}^{*}$ is applied to the unpaired pool $D_{U}$, which consists of CBCTs from the **10 new** patients' 2^nd^–15^th^ fractions (Figure 3, top‐right). We generate pseudo‐labels $\tilde{y}=f\left(x;\theta_{\text{sup}}^{*}\right)$ for each $x\in D_{U}$, yielding a pseudo‐paired dataset $D_{\text{PL}}=\left\{\left(x,\tilde{y}\right)|x\in D_{U}\right\}$.

#### 2.7.2 Distillation via Self‐Training

The final knowledge‐distilled model is trained on a combination of real and pseudo‐paired data (Figure 3, bottom‐left):

(6) $$\begin{array}{cc} L_{\text{total}} & =E_{\left(x,y\right)\in D_{L}\cup D_{L}^{\text{(new)}}}\;\left[\;L_{\text{sup}}\left(f(x;\theta_{\text{distill}}),\;y\right)\right]\\ & \;+E_{\left(x,\tilde{y}\right)\in D_{\text{PL}}}\;\left[\;L_{\text{distill}}\left(f\left(x;\theta_{\text{distill}}\right),\;\tilde{y}\right)\right]. \end{array}$$

Here, $D_{L}^{\text{(new)}}$ denotes the paired data (1^st^ fraction) from the **10 new** patients. The second term performs knowledge distillation, encouraging the model to match the supervised model's pseudo‐label $\tilde{y}$, enabling it to learn from the larger unpaired distribution.

### 2.8 Stage 3: Gradient‐Based Meta‐Guidance Post‐Training

**FIGURE 5:**
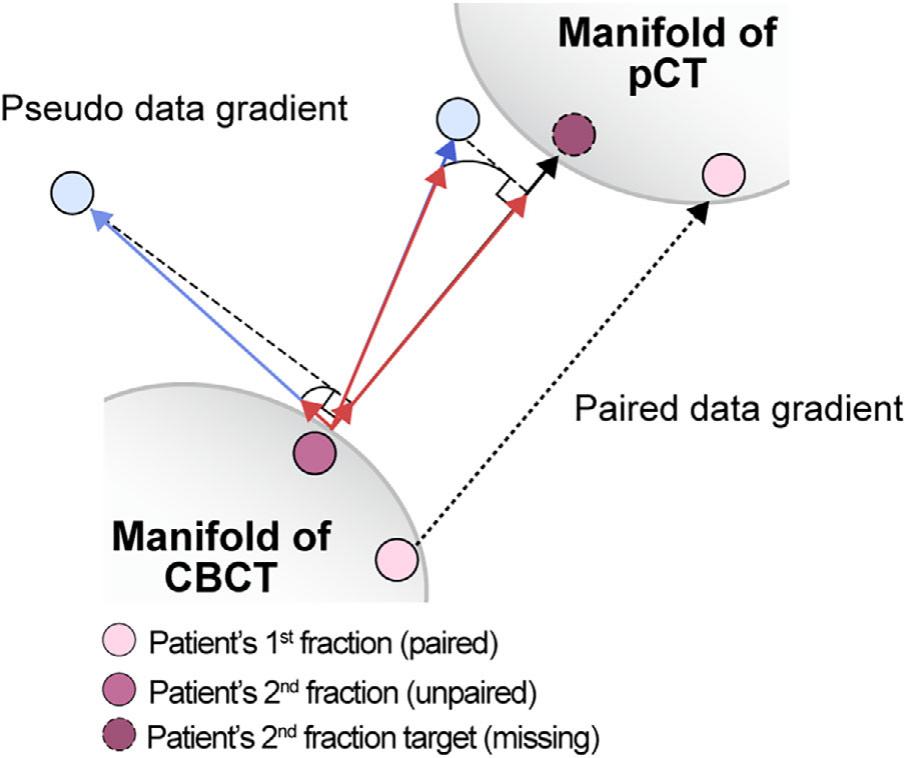
Geometric interpretation of Gradient‐Based Meta‐Guidance. For the patient's 1^st^ fraction (paired, light pink), ground truth labels exist, whereas for the 2^nd^ fraction (unpaired, dark pink), the target label is missing (dashed circle). We utilize the gradient derived from the reliable paired data (dotted arrow) as a guidance signal. By calculating the cosine similarity (dot product), we assess the direction of the pseudo‐gradients: updates that align with the guidance (red arrows) are assigned higher weights, while divergent updates (blue arrow) are suppressed. This ensures that the few paired samples effectively guide the training on abundant unpaired data to remain within the correct pCT manifold.

To stabilize the self‐training process in Stage 2, we introduce a meta‐guidance mechanism, geometrically interpreted in Figure 5. Since the label for the unpaired 2^nd^ fraction is missing, we utilize the gradients derived from the reliable 1^st^ fraction (paired data) as a guidance signal to validate the pseudo‐gradients from the unpaired fractions.

#### 2.8.1 Geometric Interpretation

For a given patient, we use the gradients derived from the reliable 1^st^ fraction (paired data, $\nabla_{\theta }L_{\text{sup}}$) as a reference to validate the gradients from the unpaired fractions (pseudo‐labeled data, $\nabla_{\theta }L_{\text{distill}}$). A naive update using incorrect pseudo‐labels can lead to hallucinations.

#### 2.8.2 Meta‐Guided Objective

We introduce a dynamic weight, w, calculated as the cosine similarity between these two gradient vectors:

(7) $$w=\max\;\left(0,\;\frac{〈\nabla_{\theta }L_{\text{distill}}\left(x\right),\;\nabla_{\theta }L_{\text{sup}}〉}{∥\nabla_{\theta }L_{\text{distill}}\left(x\right)∥\;∥\nabla_{\theta }L_{\text{sup}}∥+\varepsilon }\right),\;\varepsilon >0.$$

This weight is integrated into the final objective function:

(8) $$\begin{array}{cc} L_{\text{final}} & =E_{\left(x,y\right)\in D_{L}\cup D_{L}^{\text{(new)}}}\left[L_{\text{sup}}\left(f\left(x;\theta_{\text{distill}}\right),y\right)\right]\\ & \;+w\cdot E_{\left(x,\tilde{y}\right)\in D_{PL}}\left[L_{\text{distill}}\left(f\left(x;\theta_{\text{distill}}\right),\tilde{y}\right)\right]. \end{array}$$

This ensures that the model effectively filters out harmful updates, trusting only those pseudo‐labels consistent with the patient's known anatomy. Although calculating dual gradients introduces a marginal computational overhead per iteration, it significantly accelerates convergence stability by filtering out noisy gradients derived from incorrect pseudo‐labels. Regarding gradient computation, because memory constraints necessitate a batch size of 1 (N=1), the gradient computed per batch is inherently equivalent to the gradient computed per sample. Furthermore, we extract exact analytical gradients with respect to the entire generator parameter space using reverse‐mode automatic differentiation, without relying on any finite‐difference or partial‐layer approximations.

### 2.9 Experimental Setup

1. **Baselines**: We evaluated our proposed meta‐guided model against several key baselines to validate its performance:
  (a) **Pix2pix** ^15^: A conditional generative adversarial network (cGAN) that learns a direct mapping from source to target images. It was trained in a supervised manner using the 80‐patient paired dataset.
  (b) **CycleGAN** ^6^: An unsupervised translation model that utilizes a cycle‐consistency loss to learn from unpaired data. It was trained on all available CBCT and pCT data without explicit pairing information.
  (c) **BBDM** ^10^: A supervised Brownian Bridge Diffusion Model, representing a state‐of‐the‐art diffusion‐based method for image‐to‐image translation. It serves as a strong diffusion baseline and was trained on the 80‐patient paired set.
  (d) **Supervised**: This baseline utilizes the **identical Self‐RDB architecture** as our proposed framework but is trained exclusively on the 80‐patient paired dataset. Unlike the other supervised baselines (Pix2pix, BBDM) which use different backbones, this model serves as a direct control to isolate the specific performance gains attributable to our knowledge distillation and meta‐guidance strategies, independent of the network architecture.
2. **Evaluation Metrics**: Quantitative performance was assessed using mean absolute error (MAE), structural similarity index measure (SSIM), and peak signal‐to‐noise ratio (PSNR).
3. **Implementation Details**: Our framework was implemented using PyTorch Lightning and trained on a single **NVIDIA RTX 4090** GPU. All models were optimized using the Adam optimizer with beta parameters of (0.5,0.9). The learning rates for the generator and the discriminator were set to $1.6\times 10^{-4}$ and $1.0\times 10^{-4}$, respectively.
  The initial supervised models were trained for 100 epochs, while the final knowledge‐distilled model was fine‐tuned for 50 epochs. These exact optimization hyperparameters were kept consistent across all training stages. Key parameters for the diffusion process were set to T=10 time steps and R=2 self‐consistent recursions. The reconstruction loss was weighted by a factor of $\lambda_{\text{rec}}=0.5$. Our diffusion backbone operates as a 2D slice‐by‐slice model. To clarify the exact data volume distributed across the 99 patients, a total of 16,730 2D slices were processed. Specifically, the model utilized 4900 paired slices from the 70 patients in the legacy training cohort, 700 paired slices from the 1^st^ fraction of the 10 patients in the new cohort (serving as the meta‐guidance anchor), and 9,800 unpaired slices from their 2^nd^–15^th^ fractions for pseudo‐labeling. Finally, 1330 paired slices from the 19 patients in the held‐out legacy cohort were strictly reserved for testing. All 2D slices were resized to 256×256 pixels and trained with a batch size of 1 due to memory constraints. Regarding gradient computation under this limited batch size (N=1), the gradient computed per batch is inherently equivalent to the gradient computed per sample. Furthermore, we extract exact analytical gradients with respect to the entire generator parameter space using reverse‐mode automatic differentiation, without relying on any finite‐difference or partial‐layer approximations. To ensure optimization stability despite this constrained setting, we implemented several intrinsic stabilization measures. Rather than relying on heuristic gradient clipping or exponential moving averages, we applied an adversarial loss with an explicit gradient penalty to the discriminator. This mathematically bounds the gradient norms and robustly prevents optimization instability during meta‐guided updates. Additionally, we utilized a Cosine Annealing learning rate scheduler to facilitate smooth convergence. Furthermore, the recursive refinement mechanism within our generator iteratively dampens the stochastic noise inherent in single‐sample batches, maintaining highly stable forward predictions throughout training. To empirically verify the robustness of this configuration against initialization variance, we repeated the training process across three random seeds, which consistently demonstrated stable convergence and significant performance gains over the baseline. Additionally, to investigate the stability of the meta‐guidance weighting function, an ablation study on the numerical stabilizer ε and the max(0,·) truncation was performed; detailed quantitative results for both robustness analyses are provided in the Supplementary Material (Tables S1 and S2).
4. **Statistical Analysis**: All comparisons were conducted as *paired, two‐sided* tests. Given the non‐Gaussian distribution of image‐wise errors, we employed the **Wilcoxon signed‐rank test** to assess statistical significance. The evaluation was performed on the **19 held‐out test patients**, with quantitative metrics calculated on a **slice‐by‐slice basis** for every image. We report results as mean ± SD; tables indicate statistical significance as “(p<0.01)”, or “(n.s.)” otherwise. The significance threshold was set at α=0.01. To explicitly address potential cluster‐correlation and ensure the statistical independence of observations, we performed an additional patient‐level inference by aggregating slice‐wise metrics into a single mean value per patient (N=19). For this robust evaluation, 95% confidence intervals (CIs) were computed using a patient‐level bootstrap method with 5000 resamples (BCa method). Detailed results of this rigorous patient‐level analysis, confirming our statistical claims even under conservative testing, are provided in Table S3.

### 2.10 Use of Generative AI

During the preparation of this manuscript, the authors used Google Gemini for language editing/clarity improvement only. The authors reviewed and edited the content and take full responsibility for the accuracy and integrity of the work.

## 3 RESULTS

### 3.1 Quantitative Results

We evaluated all methods using MAE, SSIM, and PSNR; Table 2 summarizes the slice‐level results (mean ± SD). The **Proposed** model achieved the best mean performance across all three metrics (MAE 13.22 HU, SSIM 0.9516, PSNR 30.35 dB). Pairwise Wilcoxon signed‐rank tests confirmed that the **Proposed** method yields statistically significant improvements (p<0.01) over every baseline.

**TABLE 2:** Quantitative evaluation on the held‐out test set (*n* = 19 legacy patients).

Model	MAE (HU)↓	SSIM↑	PSNR (dB)↑
Input (no training)			
CBCT (Input)	42.65 ± 11.44*	0.8293 ± 0.0394*	23.64 ± 2.96*
Supervised (paired)			
Pix2pix^15^	16.25 ± 5.40^*^	0.9365 ± 0.0219^*^	29.44 ± 3.23^*^
BBDM^10^	19.01 ± 7.08^*^	0.9466 ± 0.0204^*^	29.39 ± 3.35^*^
Supervised (80 patients)	13.37 ± 5.50^*^	0.9499 ± 0.0213^*^	30.11 ± 3.48^*^
Unsupervised (unpaired)			
CycleGAN^6^	25.27 ± 18.13^*^	0.8938 ± 0.0368^*^	25.57 ± 3.23^*^
Semi/self‐training			
Knowledge‐distilled	13.37 ± 5.34^*^	0.9503 ± 0.0207^*^	30.20 ± 3.45^*^
**Proposed (w/ meta‐guidance)**	**13.22 ± 5.52**	**0.9516 ± 0.0205**	**30.35 ± 3.49**

*Notes*: All metrics (MAE, SSIM, PSNR) are reported on a **2D slice‐level basis** (mean ± standard deviation) across 1330 test slices. To clarify the training data utilized: **Supervised** baselines (Pix2pix, BBDM, and Supervised Self‐RDB) were trained exclusively on the 80‐patient paired cohort (70 legacy + 10 new 1^st^ fraction). **CycleGAN** utilized all data in an unpaired manner. The **Knowledge‐distilled** model leveraged both the 80 paired patients and the unpaired daily fractions (2^nd^–15^th^) via pseudo‐labeling. The **Proposed** method incorporates both knowledge distillation and gradient‐based meta‐guidance. Lower is better for MAE; higher is better for SSIM and PSNR.

^*^ Indicates a statistically significant difference compared to the **Proposed** method (*p* < 0.01, Wilcoxon signed‐rank test).

Specifically, compared to the strong diffusion baseline **BBDM**, the **Proposed** model reduced MAE by 5.79 HU, improved SSIM by +0.0050, and PSNR by +0.96 dB. Versus the **Supervised (80 patients)** model, the gains were a reduction in MAE of 0.15 HU, and increases of +0.0017 (SSIM) and +0.24 dB (PSNR). Furthermore, relative to the **Knowledge‐distilled** model (without meta‐guidance), we observed improvements corresponding to an MAE reduction of 0.15 HU, an SSIM increase of +0.0013, and a PSNR gain of +0.15 dB (all p<0.01). Even relative to **Pix2pix**, the **Proposed** method reduced MAE by 3.03 HU, improved SSIM by +0.0151, and PSNR by +0.91 dB, and far outperformed the unpaired **CycleGAN** baseline (e.g., MAE reduction of 12.05 HU, SSIM +0.0578, PSNR +4.78 dB; all p<0.01).

These results indicate a progressive, statistically significant improvement from a purely supervised setup to knowledge distillation, with the largest additional gains provided by our **gradient‐based meta‐guidance**. Overall, the combination of distillation with meta‐guided reweighting consistently enhances fidelity and structural agreement across all cases. Beyond image quality, the proposed framework demonstrates practical clinical feasibility in terms of computational efficiency. The model architecture is relatively lightweight, comprising approximately **46.9 M** parameters. Utilizing the DDIM sampler with 10 diffusion steps, the average inference time on a single NVIDIA RTX 4090 GPU is approximately **1.43 s** per 2D slice, resulting in a total reconstruction time of approximately **100.44 s** for a typical patient volume of 70 slices. Regarding the offline training overhead, a baseline supervised iteration requires approximately **0.33 s**. With the gradient‐based meta‐guidance activated, the dual gradient computation increases the training time to approximately **0.70 s** per iteration. While this introduces a roughly twofold increase during the training phase, it does not affect the rapid clinical inference speed and is strictly justified by the enhanced convergence stability against noisy pseudo‐labels. Furthermore, ablation studies (Table S2) confirm the stability of this mechanism. The metrics seamlessly stabilize when the numerical safeguard $\varepsilon \leq 10^{-10}$. Crucially, the max(0,·) truncation is essential for micro‐level structural preservation; its removal permits destructive gradient updates from noisy pseudo‐labels, leading to an overall performance degradation, including a statistically significant decrease in SSIM (p<0.01).

### 3.2 Qualitative Analysis

**FIGURE 6:**
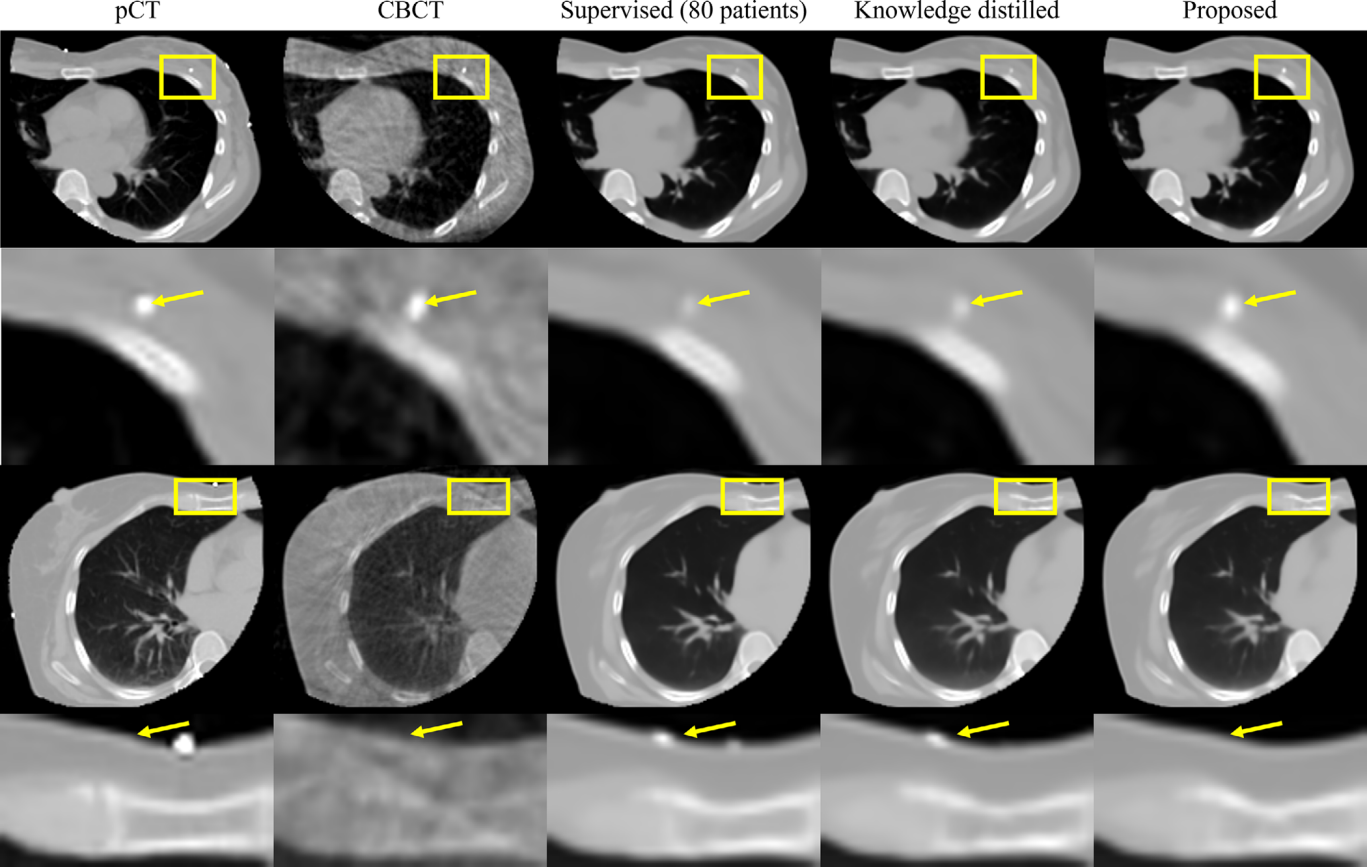
Qualitative comparison of the progressive training stages. The intermediate **Supervised** and **Knowledge‐distilled** models demonstrate a tendency to hallucinate features from the pCT, such as the fiducial marker (bottom row, yellow arrow). In contrast, our final **Proposed** model shows superior anatomical fidelity by enhancing image quality while correctly preserving the true anatomical structure of the daily CBCT, as evidenced by the well‐preserved clip structure (top row). All images are displayed with a window setting of [‐1000, 700] HU.

Figure 6 illustrates not only the progressive visual improvements but also the superior anatomical fidelity achieved by our proposed framework. As highlighted in the visual comparison, the intermediate models (**Supervised** and **Knowledge‐distilled**) demonstrate a key limitation: they tend to hallucinate features specific to the pCT prior, such as fiducial markers (bottom row, yellow arrow), which are absent in the daily CBCT. In contrast, our final model correctly adheres to the daily anatomy.

**FIGURE 7:**
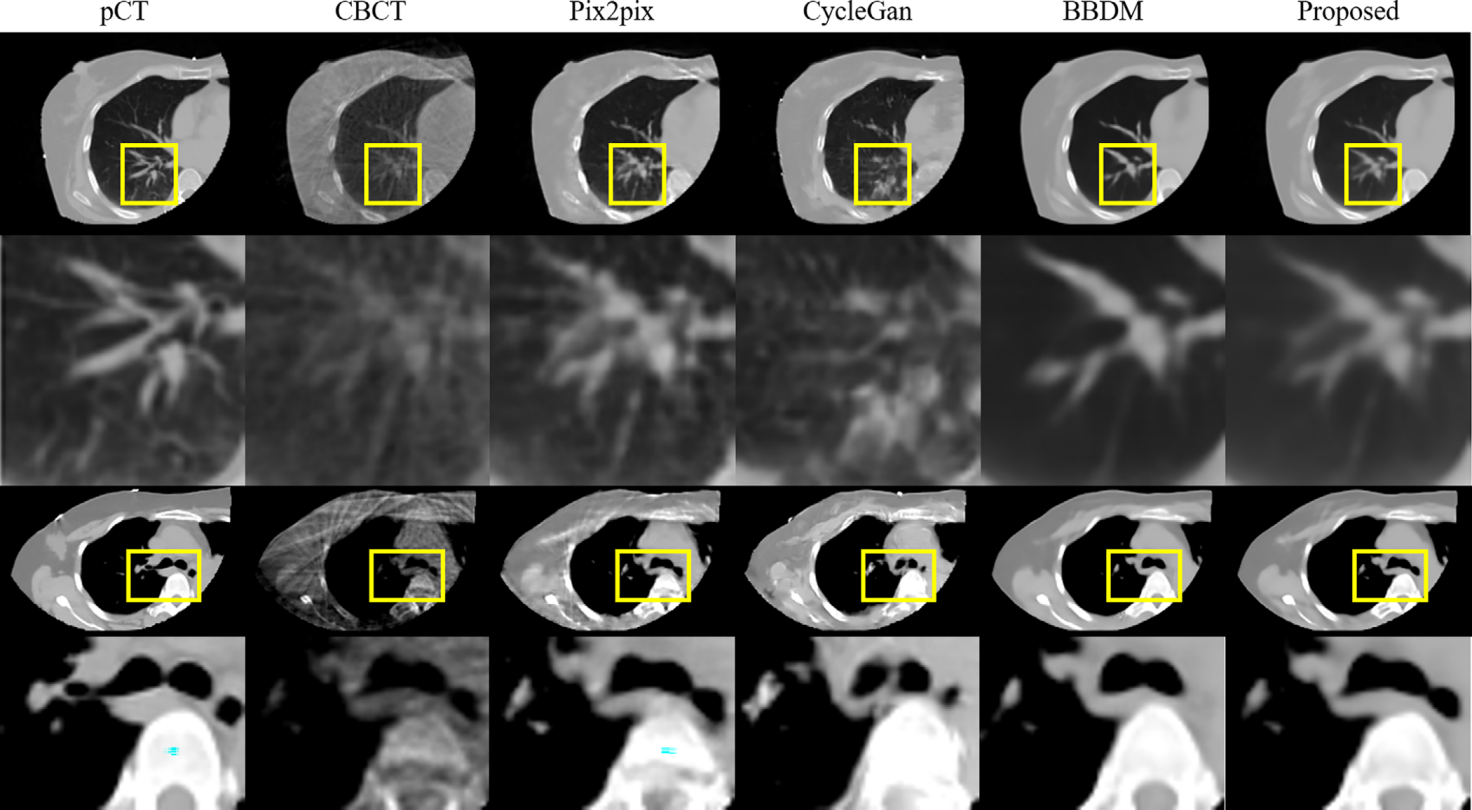
Qualitative comparison against representative baseline methods. The **Proposed** method avoids the high‐frequency artifacts of **Pix2pix**, the significant blurring of **CycleGAN**, and the over‐smoothing of fine details observed in **BBDM**. It demonstrates a superior ability to render the daily CBCT's anatomy with the high‐contrast quality of a pCT, successfully suppressing noise while preserving critical structural details, including small **lung nodules** and **cardiac vessels**. All images are displayed with a window setting of [‐1000, 700] HU.

Furthermore, Figure 7 provides a qualitative comparison against representative baseline methods from supervised, unsupervised, and diffusion‐based approaches. While **Pix2pix** shows reasonable performance, it tends to introduce subtle high‐frequency artifacts. **CycleGAN** suffers from significant blurring and a substantial loss of anatomical detail, rendering it clinically inadequate. The diffusion‐based **BBDM** produces clean images but exhibits an over‐smoothing effect, resulting in the loss of fine structures. To further evaluate the voxel‐wise fidelity and the suppression of prior leakage, detailed difference maps for all methods are provided in Figure S2 of the Supplementary Material.

In contrast, our **Proposed** model not only avoids these artifacts but also demonstrates a generally improved level of anatomical fidelity. As shown in the magnified regions, it reconstructs clinically significant features with notable accuracy that are challenging to preserve. Specifically, it reproduces the **lung nodule** and the subtle vasculature of the **cardiac vessels** with enhanced accuracy, closely matching their true anatomical appearance. Moreover, unlike other models where these fine structures are often blurred or distorted, our model preserves their original CBCT‐defined morphology with high contrast more stably. This indicates that the proposed framework not only enhances image quality but, more importantly, prevents anatomical hallucinations and more reliably captures the patient's daily anatomical variations.

As a preliminary proof‐of‐feasibility study to explore the potential clinical applicability of the proposed framework, a pilot‐scale 3D dosimetric evaluation (N=3) was conducted. Due to the highly labor‐intensive nature of manual treatment plan recalculation by clinical experts, this evaluation was inherently constrained to a limited sample size. To mitigate selection bias within this practical constraint, these three cases were randomly selected from the held‐out test cohort. By recalculating treatment plans on the synthesized CT images, as summarized in Table S5, the proposed method showed promising improvements in HU accuracy and reasonably recovered critical dosimetric parameters for both the target (PTV) and organs‐at‐risk (Heart and Lungs). While these pilot results effectively mitigate the dose calculation errors inherently observed in conventional CBCT, we temper any broad clinical claims; this serves strictly as a proof‐of‐feasibility under resource constraints, and large‐scale dosimetric validation is required to fully establish clinical utility. Detailed quantitative results and Dose‐Volume Histograms (DVH) are provided in the Supplementary Material (Table S5, Figure S3).

### 3.3 Data Efficiency Analysis

**FIGURE 8:**
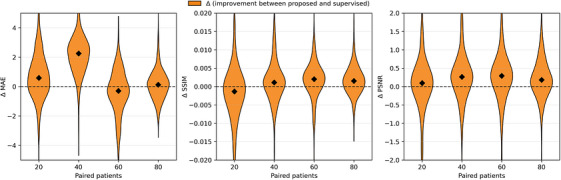
Violin plots illustrating the distributions of performance improvement (Δ) of the **Proposed** method over the **Supervised** baseline across varying paired dataset sizes (20, 40, 60, 80). The metrics include ΔMAE (scaled improvement), ΔSSIM, and ΔPSNR, calculated such that positive values indicate superior performance by the Proposed method. The dashed line at zero represents the baseline (no difference). The plots are clipped to visually emphasize the density of the distribution and the statistical significance of the improvements.

To investigate the effectiveness of our framework in data‐scarce regimes, we analyzed the *differential performance* (Δ) between the **Proposed** method and the **Supervised** baseline. Figure 8 presents violin plots of these differences across four paired data sizes (20, 40, 60, and 80 patients). In this visualization, the dashed zero line represents the baseline performance, and positive Δ values indicate an improvement achieved by the proposed method (i.e., reduced MAE, and increased SSIM/PSNR).

The distribution of Δ MAE reveals the remarkable data efficiency of our approach. In highly constrained settings (20 and 40 patients), the median improvements are substantially above the zero line, indicating that meta‐guided distillation successfully compensates for the lack of paired data to restore accurate HU intensities. As the paired dataset grows (60 and 80 patients) and the supervised baseline naturally saturates in absolute error, the Δ MAE robustly converges near zero without negative degradation. This confirms that our mechanism strictly filters out the harmful pseudo‐gradients that typically distort HU values in standard unpaired learning.

Furthermore, the Δ SSIM and Δ PSNR plots demonstrate consistent gains in image quality and structural fidelity. While structural metrics stabilize at extreme data scarcity (20 patients), the proposed method consistently shifts the distributions above the baseline for 40, 60, and 80 patients. Overall, these results confirm that the proposed framework is not only exceptionally efficient in limited‐data scenarios but also safely and robustly enhances perceptual and structural quality across all data regimes by leveraging abundant unpaired fractions.

### 3.4 Generalization Experiment On Public Dataset

To validate the generalization capability of our framework, we conducted additional experiments on a publicly available dataset from the **SynthRad 2025 challenge**, which comprises 151 head‐and‐neck cancer patients. The dataset was partitioned into a held‐out test set of **20 patients** and a training pool of 131 patients. For the **Supervised** baseline, we utilized a cohort of **75 paired patients**. For the proposed knowledge distillation framework, we expanded the training data by incorporating the remaining **56 unpaired patients** alongside the 75 paired patients, utilizing a total of 131 patients for training.

Consistent with our primary internal cohort, the SynthRad 2025 data underwent an identical two‐stage preprocessing and spatial alignment pipeline as detailed in Section 2.3. Specifically, a global 3D translation was first applied to correct bulk misalignments between the paired pCT and CBCT images. Following this rigid alignment, the volumes were cropped to match the valid FOV of the CBCT and resized to a 256×256 axial region. Subsequently, a non‐rigid registration was performed using the exact same displacement‐field‐based deformable image registration algorithm (imregdeform). The physical HU values were then explicitly clipped to a clinically relevant range of [−1000,3000] HU before being linearly normalized to approximately [−1.0,1.5]. Furthermore, to ensure a fair and rigorous evaluation protocol, all quantitative metrics (MAE, SSIM, PSNR) for this external validation were computed on a 2D slice‐by‐slice basis, strictly matching the primary evaluation methodology used for our internal dataset.

As summarized in Table 3, the inclusion of unpaired data through knowledge distillation yielded the best performance. Compared to the baseline CBCT input (MAE 122.34, SSIM 0.6961, PSNR 19.03 dB), the **Supervised** model trained with 75 paired patients significantly improved reconstruction accuracy (MAE 50.20, SSIM 0.8149, PSNR 22.20 dB; all p<0.01).

**TABLE 3:** Quantitative results on the external Public Dataset (SynthRad 2025 held‐out test set, *n* = 20).

Model	MAE (HU)↓	SSIM↑	PSNR (dB)↑
CBCT (Input)	122.34 ± 61.92^*^	0.6961 ± 0.1295^*^	19.03 ± 4.38^*^
Supervised (75 patients)	50.20 ± 29.58^*^	0.8149 ± 0.1199^*^	22.20 ± 4.04^*^
**Knowledge‐distilled**	**47.12 ± 23.16**	**0.8204 ± 0.1124**	**22.30 ± 3.97**

*Notes*: Metrics are reported on a **2D slice‐level basis** (mean ± standard deviation). The Supervised baseline was trained on 75 paired patients, whereas the **Knowledge‐distilled** model expanded the training pool by incorporating an additional 56 unpaired patients via pseudo‐labeling.

^*^ Indicates statistical significance (*p* < 0.01) compared to the **Knowledge‐distilled** model (Wilcoxon signed‐rank test).

Crucially, the **Knowledge‐distilled** model, which leveraged both the 75 paired and 56 unpaired patients, achieved the highest overall performance, reaching an MAE of 47.12 HU, SSIM of 0.8204, and PSNR of 22.30 dB. These improvements were statistically significant compared to the supervised baseline (p<0.01), confirming that the knowledge distillation strategy effectively utilizes unpaired data to enhance model robustness even on external, multi‐center datasets. It is important to explicitly clarify the scope of this external validation. Because the SynthRad 2025 dataset is cross‐sectional and lacks the subsequent longitudinal unpaired daily fractions required for patient‐specific gradient alignment, our meta‐guidance mechanism could not be applied. Therefore, rather than validating the full proposed pipeline, these external results specifically serve to validate the robustness and generalizability of our semi‐supervised knowledge distillation framework when leveraging abundant unpaired data.

Furthermore, to explicitly address non‐independence concerns inherent in slice‐wise evaluations and to align with our internal patient‐level analysis, we conducted an additional patient‐level statistical evaluation on this external test set. In this analysis, each patient's 3D volume was treated as a single independent observation (N=20). Consistent with the slice‐wise findings, the patient‐level Wilcoxon signed‐rank test confirmed that the **Knowledge‐distilled** model maintained statistically significant improvements over the supervised baseline (e.g., p<0.05 for MAE and SSIM). Detailed patient‐level aggregated results and the corresponding statistical testing are provided in Table S4.

## 4 DISCUSSION

This study proposes a semi‐supervised diffusion framework that fuses knowledge distillation with gradient‐based meta‐guidance to leverage the realistic imbalance between scarce paired data and abundant unpaired CBCT in CBCT‐to‐sCT synthesis. Beyond improving pixel‐wise fidelity, a central contribution is **stabilizing learning from noisy pseudo‐labels** by reweighting updates according to the cosine alignment between pseudo‐labeled and ground‐truth–driven gradients, thereby suppressing harmful pseudo‐updates and preserving daily anatomy.

Diffusion backbones have recently shown strong promise for CBCT→CT translation, recovering HUs while mitigating artifacts more reliably than GAN‐only pipelines. Representative reports include conditional DDPMs with improved HU accuracy and artifact reduction, as well as texture‐preserving and latent/bridge variants tailored for high‐frequency detail in radiotherapy images.^16^, ^17^, ^18^ Our Self‐RDB backbone is consistent with these trends; however, the **gains we observe arise primarily from the training scheme** rather than architectural novelty—specifically, knowledge distillation that unlocks unpaired data and gradient‐based meta‐guidance that filters noisy pseudo‐supervision. This addresses a recurrent limitation noted in challenge reports and reviews: breadth and diversity of training data, not just model design, are a dominant bottleneck for sCT quality and robustness.^19^

Learning from unpaired data is attractive yet fragile because semi‐/self‐training is sensitive to pseudo‐label noise. Prior medical‐imaging approaches introduce reliability scoring, consistency constraints, or robust pseudo‐labeling for dense prediction.^20^, ^21^Our contribution is a simple, model‐agnostic **gradient reweighting** that modulates the contribution of each pseudo‐update based on its alignment with supervised gradients. Ablations indicate particular benefits when the initial teacher is suboptimal, which is a common practical scenario.

Clinical deployment requires not only sharpness but also **structural faithfulness** to the day‐of‐treatment anatomy. Diffusion models are not immune to hallucination under distribution shifts, and recent work has proposed explicit safeguards, such as training‐free Local Diffusion to keep sampling in‐distribution and quantitative hallucination indices to audit reconstructions.^22^, ^23^ Our meta‐guidance is complementary: instead of modifying the sampler, it reduces the influence of pseudo‐labeled samples whose gradients *disagree* with supervised signals, thereby limiting the leakage of pCT‐only features into sCT. The qualitative removal of fiducials that are present in pCT but absent in CBCT aligns with this gradient‐direction view.

Several limitations remain. First, while our results show improved HU fidelity, clinical validation ultimately requires dose‐based endpoints and robust QA gates, as image similarity metrics alone do not guarantee dosimetric accuracy.^24^, ^25^ Although our supplementary dosimetric evaluation successfully demonstrated the potential to correct CBCT dose calculation errors, it was conducted as a proof‐of‐feasibility study on a randomly selected, limited sample size (N=3). Therefore, these dosimetric findings should be interpreted with caution. Large‐scale prospective ART trials with online dose recalculation are still needed to fully establish its clinical benefit. Second, to address potential distribution shifts across diverse devices and anatomies,^19^ future work could integrate patient‐specific adaptation techniques, such as lightweight LoRA fine‐tuning, to further exploit daily fractions with minimal overhead.^26^ Third, despite the overall improvements, representative failure modes such as the over‐smoothing of fine pulmonary structures, misrepresentation of high‐density surgical clips, and inaccurate bone reconstruction due to severe streak artifacts were identified (see Figures S4 and S5 in the Supplementary Material). Finally, this study is retrospective; prospective ART trials with online dose recalculation are needed to establish clinical benefit, consistent with practice‐oriented evaluations.^25^

Overall, these findings suggest that gradient‐aligned reweighting can help stabilize self‐training for CBCT‐to‐sCT synthesis when paired data are limited and pseudo‐label noise is unavoidable. Future work will focus on prospective validation with dose‐based endpoints and multi‐institutional evaluation to assess robustness across devices, anatomical sites, and acquisition protocols.

## 5 CONCLUSION

We presented a semi‐supervised diffusion framework that combines knowledge distillation with gradient‐based meta‐guidance to address the “few‐paired vs. many‐unpaired” imbalance in CBCT‐to‐sCT synthesis. By reweighting pseudo‐labeled updates via supervised‐gradient alignment, the proposed approach suppresses harmful pseudo‐supervision and improves anatomical faithfulness. On a 99‐patient breast cancer cohort, it achieved statistically significant improvements over representative baselines, and experiments on a public dataset further supported the benefit of leveraging unpaired data for robustness.

## CONFLICT OF INTEREST STATEMENT

The authors declare that they have no relevant conflicts of interest to disclose.

## Supporting information

Supporting Information

## Data Availability

The internal breast cancer dataset analyzed in this study is not publicly available due to patient privacy regulations. The external validation dataset is publicly available through the SynthRad 2025 challenge. Source code for the proposed framework will be made publicly available via a GitHub repository upon acceptance of this manuscript.
