# Supplementary material for: Knowledge‐distilled diffusion models for improving cone‐beam CT image quality with meta‐learning under imbalanced data
Source: Med Phys. 2026 Jul 31;53(8):e70530. doi: 10.1002/mp.70530 (PMC13425450; doi:10.1002/mp.70530)
Supplement: Supplementary file 1 — Supporting Information [file MP-53-0-s001.zip › Table.docx]

**Table S1**: Robustness evaluation across three different random seeds. The models were trained with a batch size of 1. The results demonstrate that the Proposed model is highly stable to initialization variance, consistently achieving superior structural fidelity (higher SSIM, PSNR) and lower quantitative error (lower MAE) compared to the baseline Teacher model across all seeds. (Reported values indicate the mean on the held-out test set).

| **Random Seed** | **Model** | **MAE (HU) ↓** | **SSIM ↑** | **PSNR (dB) ↑** |
| --- | --- | --- | --- | --- |
| **Seed random1** | Teacher (70 pts) | 13.98 | 0.9493 | 30.08 |
|  | Proposed | **13.22** | **0.9516** | **30.35** |
| **Seed random2** | Teacher (70 pts) | 14.04 | 0.9500 | 30.13 |
|  | Proposed | **12.92** | **0.9516** | **30.30** |
| **Seed random3** | Teacher (70 pts) | 15.09 | 0.9500 | 30.10 |
|  | Proposed | **13.71** | **0.9510** | **30.34** |
| **Average ± Std** | Teacher (70 pts) | 14.37 ± 0.62 | 0.9498 ± 0.0004 | 30.10 ± 0.03 |
|  | Proposed | **13.28 ± 0.40** | **0.9514 ± 0.0003** | **30.33 ± 0.03** |

**Table S2**: Ablation study on the sensitivity and stability of the meta-guidance weighting function (Eq. 7). The asterisk (^∗^) indicates a statistically significant difference compared to the Proposed model (p < 0.01, Wilcoxon signed-rank test), while (n.s.) denotes not significant.

| **Component** | **Configuration** | **MAE (HU) ↓** | **SSIM ↑** | **PSNR (dB) ↑** |
| --- | --- | --- | --- | --- |
| **Baseline** | **Proposed (ϵ = 10^−10^)** | **13.22 ± 5.52** | **0.9516 ± 0.0205** | **30.35 ± 3.49** |
| **Sensitivity** | Ablation (ϵ = 10^−9^) | 13.19 ± 5.30 | 0.9514 ± 0.0216 | 30.32 ± 3.44 |
|  | Ablation (ϵ = 10^−11^) | 13.22 ± 5.52 | 0.9516 ± 0.0205 | 30.35 ± 3.49 |
|  | Ablation (ϵ = 10^−12^) | 13.22 ± 5.52 | 0.9516 ± 0.0205 | 30.35 ± 3.49 |
| **Truncation** | Without max(0, ·) | 13.42 ± 5.58^∗^ | 0.9482 ± 0.0232^∗^ | 30.09 ± 3.51^∗^ |

**Table S3**: Patient-level aggregated quantitative results (N = 19). Results are formatted as Mean ± SD [95% Bootstrap CI]. Lower is better for MAE; higher for SSIM and PSNR. The asterisk (^∗^) indicates a statistically significant difference compared to the **Proposed** method (p < 0.01, Wilcoxon signed-rank test).

| **Model** | **MAE (HU)↓** | **SSIM↑** | **PSNR (dB)↑** |
| --- | --- | --- | --- |
| *Input (no training)* |  |  |  |
| CBCT (Input) | 42.65 ± 8.73^∗^ [39.15, 47.04] | 0.8293 ± 0.0265^∗^ [0.8163, 0.8404] | 23.64 ± 1.77^∗^ [22.80, 24.40] |
| *Supervised (paired)* |  |  |  |
| Pix2pix | 16.25 ± 3.55^∗^ [15.10, 18.76] | 0.9365 ± 0.0128^∗^ [0.9304, 0.9420] | 29.44 ± 1.84^∗^ [28.23, 30.06] |
| BBDM | 19.01 ± 3.47^∗^ [17.84, 21.29] | 0.9466 ± 0.0128^∗^ [0.9406, 0.9522] | 29.39 ± 1.94^∗^ [28.20, 30.08] |
| Supervised (80 patients) | 13.37 ± 3.72 (n.s.) [12.20, 16.14] | 0.9499 ± 0.0128^∗^ [0.9435, 0.9552] | 30.11 ± 2.02^∗^ [28.77, 30.79] |
| *Unsupervised (unpaired)* |  |  |  |
| CycleGAN | 25.27 ± 8.05^∗^ [22.95, 31.66 | 0.8938 ± 0.0209^∗^ [0.8797, 0.9003] | 25.57 ± 1.59^∗^ [24.67, 26.16] |
| *Semi/self-training* |  |  |  |
| Knowledge-distilled | 13.37 ± 3.72 (n.s.) [12.22, 16.17] | 0.9503 ± 0.0128^∗^ [0.9438, 0.9556] | 30.20 ± 2.06^∗^ [28.80, 30.88] |
| **Proposed (ours)** | **13.22 ± 3.68 [12.05, 15.90]** | **0.9516 ± 0.0122 [0.9456, 0.9568]** | **30.35 ± 2.04 [28.96, 31.03]** |

**Table S4**: Patient-level aggregated quantitative results on the external SynthRAD 2025 dataset (N = 20). Results are formatted as Mean ± SD [95% Bootstrap CI]. The asterisk (^∗^) indicates a statistically significant difference compared to the Knowledge-distilled model (p < 0.05, Wilcoxon signed-rank test).

| **Model** | **MAE (HU)↓** | **SSIM↑** | **PSNR (dB)↑** |
| --- | --- | --- | --- |
| *Input (no training)* |  |  |  |
| CBCT (Input) | 122.34 ± 59.73^∗^ [96.34, 147.65] | 0.7268 ± 0.1164^∗^ [0.6722, 0.7730] | 23.70 ± 3.65^∗^  [22.21, 25.35] |
| *Supervised (paired)* |  |  |  |
| Supervised (75 pts) | 50.20 ± 24.51^∗^ [42.56, 66.44] | 0.8414 ± 0.0975^∗^ [0.7886, 0.8763] | 26.69 ± 2.67 (n.s.) [25.43, 27.77 |
| *Semi-supervised* |  |  |  |
| Knowledge-distilled | **47.12 ± 19.78 [40.17, 58.04]** | **0.8479 ± 0.0894 [0.8010, 0.8811]** | **26.81 ± 2.68**  **[25.59, 27.93]** |

**Table S5**: Quantitative dosimetric results averaged over the patient cohort (N = 3). To highlight the most impactful clinical improvements, we focused on physical density (HU MAE), target dose coverage and hotspot metrics (PTV D_max_, D_98_, V_95_), and critical safety metrics for OARs where CBCT typically exhibits significant deviations (Heart D_max_, V_5_, V_10_; Lungs D_max_, V_20_). All values are expressed as Mean ± Standard Deviation.

| **ROI** | **Metric** | **Reference (pCT)** | **Original CBCT** | **Proposed pCT** |
| --- | --- | --- | --- | --- |
| **Total PTV** | HU MAE (HU) | – | 94.05 ± 19.61 | **14.75 ± 3.74** |
|  | D_max_ (Gy) | 49.58 ± 0.11 | 49.53 ± 0.18 | **49.53 ± 0.14** |
|  | D_98_ (Gy) | 38.38 ± 0.61 | 38.22 ± 0.77 | **38.35 ± 0.72** |
|  | V_95_ (%) | 20.67 ± 4.17 | 20.61 ± 4.21 | **20.62 ± 4.16** |
| **Heart** | HU MAE (HU | – | 96.27 ± 19.48 | **11.31 ± 2.99** |
|  | D_max_ (Gy) | 9.43 ± 8.34 | 10.38 ± 10.35 | **9.48 ± 8.51** |
|  | V_5_ (%) | 1.16 ± 2.00 | 1.37 ± 2.30 | **1.18 ± 2.05** |
|  | V_10_ (%) | 0.10 ± 0.18 | 0.16 ± 0.27 | **0.14 ± 0.24** |
| **Lungs** | HU MAE (HU) | – | 41.49 ± 8.86 | **21.75 ± 10.05** |
|  | D_max_ (Gy) | 38.98 ± 3.74 | 39.24 ± 3.59 | **38.46 ± 3.37** |
|  | V_20_ (%) | 2.90 ± 0.72 | 3.03 ± 0.61 | **2.94 ± 0.59** |
| **Global** | Gamma 3mm/3% (%) | 100.00 | 92.79 ± 2.54 | **93.08 ± 2.72** |
